# Immune and Inflammation in Acute Coronary Syndrome: Molecular Mechanisms and Therapeutic Implications

**DOI:** 10.1155/2020/4904217

**Published:** 2020-08-18

**Authors:** Haiming Wang, Zifan Liu, Junjie Shao, Lejian Lin, Min Jiang, Lin Wang, Xuechun Lu, Haomin Zhang, Yundai Chen, Ran Zhang

**Affiliations:** ^1^Department of Cardiology, The First Medical Center of Chinese PLA General Hospital & Chinese PLA Medical School, Beijing 100853, China; ^2^The First Clinical Medical College of Inner Mongolia Medical University, Hohhot 010059, China; ^3^Department of Cardiology, The Eighth Medical Center of Chinese PLA General Hospital, Beijing 100091, China; ^4^Department of Hematology, The Second Medical Center of Chinese PLA General Hospital & Chinese PLA Medical School, Beijing 100853, China

## Abstract

Acute coronary syndrome (ACS) is a major cause of acute death worldwide. Both innate and adaptive immunity regulate atherosclerosis progression, plaque stability, and thrombus formation. Immune and inflammation dysfunction have been indicated in the pathogenesis of ACS. The imbalance in the proatherogenic and antiatherogenic immune networks promotes the transition of plaques from a stable to unstable state and results in the occurrence of acute coronary events. The residual inflammatory risk (RIR) has received increasing attention in recent years, and lowering RIR has been expected to improve the outcomes of ACS patients. The CANTOS, COLCOT, and LoDoCo trials verified the benefits of reducing cardiovascular events using anti-inflammation therapies; however, most of the other studies focusing on lowering RIR produced negative or contradicting results. Therefore, restoring the balance in autoimmune regulation is essential because proatherogenic and antiatherogenic immunomodulatory effects are equally important in the complex human immune network. In this review, we summarized the recent evidence of the roles of proatherogenic and antiatherogenic immune networks in the pathogenesis of ACS and discussed how immune and inflammation contribute to atherosclerosis progression, plaque instability, and adverse cardiovascular events. We also provide a “from bench to bedside” perspective of a novel and promising personalized strategy in RIR intervention and therapeutic approaches for the treatment of ACS.

## 1. Introduction

Acute coronary syndrome (ACS) refers to a complex clinical syndrome that includes a spectrum of entities including unstable angina, ST segment elevation myocardial infarction (STEMI), and non-ST segment elevation myocardial infarction (NSTEMI). The accumulating evidence has implicated an inflammatory process in the pathogenesis of ACS that involves local immune cells in coronary arteries generating inflammatory factors that promote thrombus formation [1, 2]. Although nonatherosclerotic factors can also contribute to ACS, the most common cause of ACS is atherosclerotic plaque rupture or erosion with subsequent thrombus formation. Nearly 60% of ACS patients have a high level of high-sensitivity C-reactive protein (hsCRP) (>2.0 mg/L), a biomarker of systemic inflammation and a predictive factor of high cardiovascular mortality, which is defined as the residual inflammatory risk (RIR) in these patients [3]. Atherosclerosis has been recognized as a chronic inflammatory disorder characterized by dysfunctional immune-inflammation involving interactions between immune cells (macrophages, T lymphocytes, and monocytes) and vascular cells (endothelial cells, smooth muscle cells) [4]. Systemic or local inflammation promotes coronary thrombus formation. Both innate and adaptive immune responses contribute to atherosclerosis and its thrombotic complications in ACS through complex interactions between atherosclerosis, innate immunity, and inflammation [5]. The CANTOS trial (The Canakinumab Anti-inflammatory Thrombosis Outcomes Study) targeted interleukin-1*β* (IL-1*β*) innate immunity with canakinumab in patients with a history of ACS and hsCRP ≥ 2.0 mg/L and reported significantly attenuated systemic inflammation indicated by hsCRP and improved clinical outcomes independent of lipid metabolism [6]. This study demonstrated that coronary RIR could be successfully treated by inhibiting IL-1*β* with canakinumab and raised a potential implication of anti-inflammatory therapy in ACS patients. This article reviews recent advances in the understanding of immune-mediated inflammation in the pathogenesis of ACS and discusses its implications for ACS management strategies.

## 2. Current Status of Inflammatory Risk in ACS

### 2.1. Immune-Mediated Inflammation and the Pathogenesis of ACS

The molecular mechanisms of atherosclerosis are cholesterol deposition and immune cell aggregation in the arterial wall. Innate and adaptive immune cells with both proinflammatory and anti-inflammatory effects regulate subsequent atherosclerosis progression. The transition from stable to unstable plaques with subsequent rupture or erosion and thrombus formation contribute to ACS [2]. The link between immune-mediated inflammation and ACS is complex, and the underlying mechanisms of ACS are not fully understood. The pathogenesis of ACS can be divided into plaque rupture with systemic inflammation and red thrombus, plaque rupture with low systemic inflammation, plaque erosion with white thrombus, and ACS without epicardial coronary artery thrombus or stenosis [2]. Usually, at the sites of plaque rupture, activated macrophages and T cells secrete cytokines that trigger a self-perpetuating vicious circle reaction, eventually leading to the fragile and thin fibrous cap as well as the accumulation of a central lipid core [7, 8]. Therefore, systemic and local inflammatory responses are important causes of ACS. Plaque rupture with low systemic inflammation was characterized by no accumulation of macrophages in ruptured plaques and low-grade systemic inflammation. This kind of plaque rupture is caused by psychological stress or local vascular wall stress. Similarly, coronary plaque erosion with white thrombus is not related to macrophage-mediated inflammation, but to platelet aggregation. Although coronary plaque erosion is considered to have no explicit relationship to systemic inflammation, immune cells, and inflammatory factors have been shown to be involved in this process [2]. ACS without epicardial coronary artery thrombus or stenosis may arise from coronary vasospasm or microvascular disease.

Immune-mediated inflammatory disorders, including rheumatic diseases, inflammatory bowel disease (IBD), rheumatoid arthritis (RA), and systemic lupus erythematosus, are also closely related to acute cardiovascular events independent of traditional cardiovascular risk factors. ACS is very common in young women with IBD who often show a high level of CRP [9]. Therefore, immune-mediated inflammation but not the traditional risk factors is the main cause of these acute cardiovascular events. However, a retrospective cohort study of 300 IBD patients without traditional risk factors in North Shore University Hospital failed to identify the relationship between IBD and acute cardiovascular events [10]. It is possible that the inflammation level is not enough to cause plaque rupture. However, the activity of inflammation is more important than the duration of inflammatory disorders. RA is associated with a higher incidence of premature cardiovascular events and a twofold increase in the incidence of ACS [11, 12]. RA patients who respond well to anti-inflammatory medication have a decreased risk of future ACS, suggesting that inflammation induces ACS [13]. Rheumatic diseases contribute to events through specific systemic inflammation in the absence of commonly known cardiovascular risk factors. This cause-and-effect relationship is not limited to rheumatic diseases and ACS. Any disorders involving systemic inflammation may damage coronary arteries and subsequently induce ACS [14]. This immunological pathogenesis may provide a theoretical basis for potential clinical applications and interventions.

### 2.2. RIR: A Gap in the Management of ACS

The CANTOS trial showed that canakinumab was associated with a 15% reduction in major adverse cardiovascular events (MACEs), indicating the importance of anti-inflammatory therapy in the management of ACS residual risks [6, 15]. In the FOURIER (Further Cardiovascular Outcomes Research with PCSK9 Inhibition in Subjects With Elevated Risk) trial, patients with low − density lipoprotein cholesterol (LDL − C) < 30 mg/dL but elevated hsCRP showed a higher incidence of MACE than patients with LDL − C > 100 mg/dL, despite the use of proprotein convertase subtilisin-kexin type 9 (PCSK9) inhibitors [16]. In the post hoc analysis of the SPIRE (Studies of PCSK9 Inhibition and the Reduction in Vascular Events) trials, stable outpatients receiving high-intensity lipid-lowering therapies still showed a 60% increased risk of future cardiovascular events when hsCRP levels exceeded 3 mg/L [17]. This evidence suggested that RIR still exists in ACS patients who received guideline-based medication with intensive lipid-lowering therapy and lifestyle modification [3]. Therefore, the pathogenesis and management of ACS should also focus on reducing RIR beyond antiplatelet and lipid-lowering therapy in the future [3, 6].

## 3. Immune and Inflammation in the Pathogenesis of ACS

**Figure 1:**
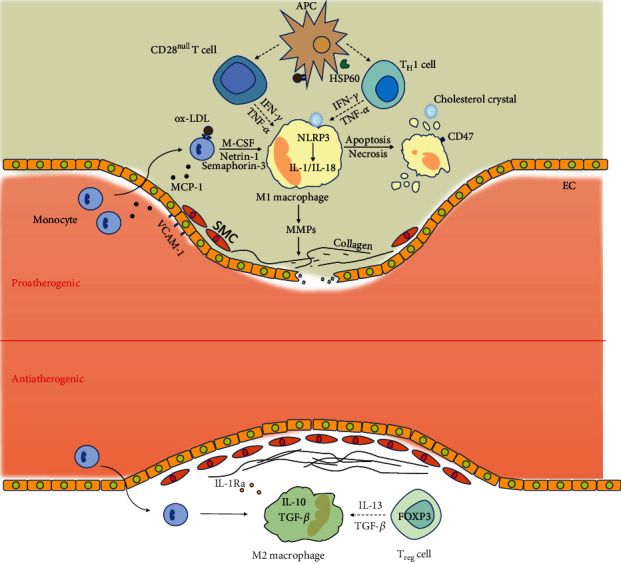
Immune and inflammation pathways in the pathogenesis of acute coronary syndrome.

The accumulating evidence has demonstrated that vascular inflammation plays pivotal roles in the pathogenesis of ACS, and thus ACS is considered an inflammation-related disease. Following systemic or local inflammatory activation, endothelial cells enhance the attachment and migration of T lymphocytes and macrophages into the arterial wall via upregulated adhesion molecules. During this process, both the proatherogenic and antiatherogenic immune networks are activated (Figure 1). Once the balance is disturbed by various traditional cardiovascular risk factors, ACS occurs and develops into acute coronary events.

### 3.1. Proatherogenic Immune Networks in ACS

#### 3.1.1. Proatherogenic Roles of Innate and Adaptive Immunity in Plaque Rupture

The innate immune system is the first barrier for human self-protection that activates nonspecific immune cells to respond to pathogens [18], and immune cells are restricted to enter into the vascular endothecium under normal physiological conditions [19]. The intimal lipid particles in the artery wall promote the production of selectins, monocyte chemoattractant protein-1 (MCP-1), and vascular cell adhesion molecule-1 (VCAM-1) in endothelial cells, which provide monocytes and other inflammatory cells with specific stimuli and anchors [15, 19]. Macrophages are associated with atherosclerosis progression and plaque rupture. After recruitment, monocytes continue to propagate and differentiate into proinflammatory macrophages (M1 type) stimulated by monocyte-colony-stimulating factor (M-CSF) [8]. Platelets are not only important in thrombus formation, but also also contribute to atherosclerotic inflammation [20]. Platelets can induce monocyte migration and recruitment to form plaques and also mediate macrophage polarization to the M1 type [20]. Netrin-1 and semaphorin-3 block binding to CCL19 and CCL21 and prevent lipid-phagocytic macrophages from migrating out of diseased lesions [8, 21]. The degree of macrophage efflux increases following the intentional lowering of netrin-1 concentrations in LDLR^−/−^ mice [22]. Macrophages within the atherosclerotic plaque undergo apoptosis or necrosis [8] and are not effectively removed by efferocytosis [23]. CD47, an antiphagocytic signal molecule that helps evade the autoimmune system, cannot be cleared by efferocytosis [24]. CD47 is upregulated in the lipid core of atherosclerotic plaques [25] and blocks phagocytic function in plaques [26], which may be involved in the transition from stable to unstable plaques.

A thin fibrous cap is common in unstable plaques [27] and is associated with the impaired metabolism of interstitial collagens [28]. Typically, tensile interstitial collagen is resistant to degradation by a majority of proteases except for matrix metalloproteinases (MMPs) [29]. Macrophages are one of the primary sources of synthetic MMPs [30]. The enhanced activity or abundance of MMPs promotes the breakdown of the arterial extracellular matrix [8] and plaque rupture [31]. A recent study showed that inhibiting macrophage-derived MMP-13 in mice increased the number of interstitial collagens and subsequently stabilized plaques [29].

The adaptive immune system also contributes to plaque rupture. T lymphocyte subsets with distinct effector roles have multiple inflammatory functions [7]. Both helper T (T_H_) CD4^+^ cells, especially T_H_1 cells and cytotoxic T (T_C_) CD8^+^ cells, are found in atherosclerotic plaques [7]. Stimulated by dendritic cells binding with oxidized LDL or heat shock protein (HSP) 60, T_H_1 cells promote interferon-*γ* (IFN-*γ*) production, which impairs the production of interstitial collagens in vascular smooth muscle cells (VSMCs) [19, 29, 32]. T_H_1 cells and macrophages render the thin fibrous cap susceptible to ACS. CD28^null^ T cells are a subtype of T_H_ cells that are much more common in patients with ACS than in patients with stable angina, and they accelerate endothelial cell injury through the production of granzyme B and perforin [7, 33]. CD28^null^ T cells are positively correlated with the incidence of ACS [34, 35]. As for the pathogenetic mechanism, CD28^null^ T cells release excessive IFN-*γ* and TNF-*α* and resist apoptosis, which contributes to plaque vulnerability in ACS [7, 33, 35, 36]. The roles of T_C_ cells and other T_H_ cell subtypes, including T_H_2, T_H_9, T_H_22, and follicular helper T (T_FH_) cells, in ACS remain to be established [36].

The nucleotide-binding leucine-rich repeat-containing pyrin receptor 3 (NLRP3) inflammasome is a critical component of the innate immune system and triggers the immune cell release of inflammatory cytokines [19, 37]. In ACS, activated NLRP3 inflammasome produces bioactive IL-1*β* and IL-18 through activated caspase 1 in patients with atherosclerosis [16]. Both IL-1*β* and IL-18 destabilize the plaque by upregulating VCAM, thereby inducing T cell differentiation and promoting downstream proinflammatory reactions [19, 38]. IL-6 mobilizes hepatocytes to synthesize acute phase reactants containing fibrinogen, plasminogen activator inhibitor-1, and CRP [8, 19] and is associated with the evolution of ACS [39]. CRP interacts with Fc transport receptors and subsequently promotes proinflammatory cytokine production and aggravates the local proatherogenic state [19, 40]. In addition to interleukin-like proatherogenic cytokines, TNF-*α* released by macrophages induces vascular endothelial dysfunction to promote thrombosis and consistently upregulate CD47 [25, 41]. The excessive necrotic phagocytic debris stimulates Toll-like receptors (TLRs) via damage-associated molecular patterns (DAMPs) to potentiate the inflammatory elaboration [8]. Therefore, a positive feedback loop in which all proatherogenic cytokines exacerbate endothelial injury in various ways develops, and consequently, an increasing number of immune cells are attracted to the atheroma lesion [8].

#### 3.1.2. Inflammatory Mechanism of Plaque Erosion

Unlike plaque rupture, macrophages, and T lymphocytes are seldom associated with plaque erosion [2]. Eroded lesions harbor numerous glycosaminoglycans and proteoglycan instead of a large lipid core and interstitial collagens [2, 29]. Therefore, the molecular mechanism of plaque rupture and erosion differ in phenotypical and functional aspects, which can explain why plaque erosion is not accompanied by detectable systemic inflammation. In general, superficial plaque erosion is mainly initiated by endothelial cell damage caused by various factors, including hemodynamic disturbance, oxidative stress, and TLR activation mediated by hyaluronic acid [2, 42]. Injured endothelial cells covering the plaque progressively detach from the basement membrane. The platelets subsequently recruit to the denuded lesions and release granules to chemotactically attract a large number of neutrophils [2]. However, neutrophils do not settle on the surface of the plaque but exhibit neutrophil extracellular traps (NETs) via apoptosis, which further promotes local thrombosis [43]. Plaque erosion with thrombus shows a higher level of leukocyte myeloperoxidase in ACS postmortem specimens [2, 44].

### 3.2. Antiatherogenic Immune Networks in ACS

Antiatherogenic immune networks counteract the progressive proatherogenic infiltrates in the pathogenesis of ACS, which mediates a waxing and waning inflammatory environment. Some macrophages differentiate into antiatherogenic subsets (M2 type) to eliminate excessive autoimmune attacks [45]. M2 macrophages are more effective at removing necrotic cellular debris, promoting angiogenesis, and producing IL-10 and TGF-*β* [8, 45]. IL-10 induces more macrophages toward M2 subtype, and TGF-*β* controls the proliferation of immune cells, thereby restricting the proatherogenic responses [8, 46]. There are significantly more M2 macrophages in advanced plaques than M1 macrophages, which is associated with the stimulation and amplification of the proinflammatory state [8]. IL-1*β* provokes a competitive reaction with IL-1 receptor antagonists (IL-1Ra) in the atheromatous plaque, leading to spontaneous arterial inflammation [19]. In ACS patients who have a higher level of IL-1Ra than those with stable CAD, IL-1Ra appears to be associated with plaque activity and elicits a potential protective effect on the impaired myocardium [19, 47].

Recently, adaptive immunosuppressed cells have received great attention. Regulatory T (T_reg_) cells are a subtype of immunosuppressed T cells with positive FOXP3, CD20, and CTLA4 expressions that suppress antigen-presenting cells, naive and effector T cells, and natural killer (NK) cells [36]. In addition, T_reg_ cells can delay or even reverse the progression of atherosclerosis [48–50]. Once activated, T_reg_ cells release antiatherogenic cytokines, including IL-13 and TGF*β*, to promote plaque stabilization [51]. In addition, T_reg_ cells can remodel plaques by increasing the number of M2 macrophages, reconstructing damaged fibrous caps, and inhibiting the proliferation of proatherogenic T cells [52]. The preference of T_reg_ cell differentiation is enhanced in early hypercholesterolemia [53]. However, in advanced atherosclerotic lesions, T_reg_ cells selectively express T-bet, which changes their phenotype from an antiatherogenic inflammatory one to a proatherogenic state [54]. As the number of T_reg_ cells in plaques decreases, the self-protective antiatherogenic function gradually declines [55]. Compared with the general population, patients with ACS have a lower number of T_reg_ cells [56, 57]. Therefore, it is reasonable to speculate that one of the possible causes of unstable plaque is the excessive consumption of T_reg_ cells and changes in the direction of T cell differentiation [36]. FOXP3, a key regulatory molecule of T_reg_ cells, is important in the pathological differentiation of T_reg_ cells [58], and T_reg_ cells are prone to exhibit proatherogenic properties in the absence of stable FOXP3 expression [36], but the detailed mechanism of this process remains unclear. Clarifying this mechanism is of great significance to understand the antiatherogenic progression of ACS through stabilizing the FOXP3 expression to inhibit the pathological differentiation of T_reg_ cells.

## 4. Better Attenuation of Coronary RIR Lowers the Risks of ACS

Given that a large part of ACS is a systemic inflammation-related disease in which both innate and adaptive immune systems are involved, RIR may be even common besides cholesterol risk [59]. Even in these patients who received successful percutaneous coronary intervention (PCI) to relieve myocardial ischemia, persistently high RIR was common and positively correlated with one-year MACE [60]. Although this retrospective cohort study could not exclude the risk of traditional confounding cardiovascular factors, it suggests that stratifying patients based on hsCRP levels is beneficial for the prediction of cardiovascular disease risk [60]. Currently, hybrid positron emission tomography imaging, coronary computed tomography angiography (CCTA), and biomarkers of gut microbiota have been used to stratify cardiovascular risks [15, 61]. Hybrid positron emission tomography imaging is useful in underlying cardiovascular inflammatory states [62]; however, its extensive clinical application is limited because of the prolonged exposure of patients to radiation and its expensive cost. CCTA, a common clinical noninvasive method for screening obstructive CAD, can identify morphological traits of high-risk plaques and provide indirect evidence of coronary inflammation [15, 63]. The gut microbiota can release proatherogenic metabolites and induce trimethylamine N-oxide production to induce specific inflammatory responses in epicardial adipose tissue [61, 64], which provides certain valuable information in ACS prediction. However, whether or not the intestinal microbiota can be widely used as a biomarker for RIR stratification requires further investigation.

Regarding the lack of currently available methods for RIR identification, it is difficult to identify the ACS patients that require adjuvant anti-inflammatory treatment. Therefore, it is necessary to propose a novel, efficient, and cost-effective predictive model that does not only focus on the narrow degree of the regional lumen [15]. The persistently high level of hsCRP suggests that RIR continues to drive plaque progression [19], and hsCRP is an ideal biomarker to evaluate cardiovascular events because it can reflect the active state of the inflammatory cascade [65]. A newly established risk prediction score based on hsCRP levels better identifies and stratifies high-risk ACS patients than traditional risk scores, and the detection of hsCRP is a promising approach in guiding therapeutic strategies and evaluating curative effects [3]. The perivascular fat attenuation index (FAI) is an emerging biomarker of vascular inflammation based on noninvasive CCTA that can be used as a direct metric of inflammation [15]. When the plaque is in an active state of inflammation, some inflammatory factors, including IL-6 and TNF-*α*, promote the breakdown of PVAT [15, 66]. Clinical trials have found that patients with high FAI in the proximal right coronary artery were more prone to ACS [15, 67]. Furthermore, the combination of FAI and anatomical features of high-risk plaques in CCTA can be used to identify excessive RIR [15]. Therefore, the integration of CRP- or FAI-assisted CCTA to traditional cardiovascular risk scores provides accurate stratification, improves the predictive value for high-risk ACS, and paves the way for future precision medicine.

## 5. Therapeutic Approaches Targeting Immune and Inflammation in ACS

The imbalance between the proatherogenic and antiatherogenic immune networks is an essential component in the pathogenesis of ACS, although controversies regarding the contribution of inflammation to plaque erosion still exist. In addition to potent lipid-lowering manipulations, efforts to eliminate this imbalance may be a promising therapeutic strategy for ACS. Currently, although some clinical trials on anti-inflammatory treatments in ACS have obtained satisfactory endpoints; most of them have failed to complete the translation from the theoretical insight into quantifiable benefits (Table 1).

### 5.1. Targeting Proatherogenic Immune Modulation to Inhibit Inflammation in ACS

Given that proatherogenic immune networks dominate the pathogenesis of ACS, targeting proatherogenic immune modulation to inhibit inflammation in ACS is promising in this regard. The most successful study in targeting anti-inflammatory therapy in ACS was the CANTOS trial, which randomized 10,061 patients with previous MI and hsCRP ≥ 2.0 mg/L who were identified as having RIR [6]. Canakinumab (150 mg every 3 months), a monoclonal antibody targeting IL-1*β*, significantly decreased MACE and inflammatory biomarkers independent of aggressive cholesterol control [6]. This clinical trial demonstrated the importance of neutralizing the main proatherogenic pathway involving IL-1*β* to IL-6 to CRP [16]. However, immunosuppressive drugs may result in secondary infections because of their roles against the host defense system, as evidenced in the CANTOS trial in which canakinumab therapy led to an increase in infectious death [6]. Hence, early detection and effective antibiotic treatment can help counteract the risk of infection when immunosuppressive drugs are used. Methotrexate can reduce the amount of proinflammatory cytokines and limit the storage of cholesterol in macrophages [12, 19]. The Cardiovascular Inflammation Reduction (CIRT) trial is a randomized clinical trial investigating whether low-dose methotrexate can alleviate MACEs in type 2 diabetes or metabolic syndrome patients with recent ACS. The results showed a negligible effect on the level of IL-1*β* and IL-6 and the number of MACE [68]. Similarly, ACS patients administered with the IL-6 inhibitor tocilizumab showed a reduced level of troponin and inflammatory markers in the ASSessing the Effect of Anti-IL-6 Treatment in MI (ASSAIL-MI) trial [16, 69, 70], which was designed to assess the effects of tocilizumab on ischemia reperfusion injury in patients with ST-elevation myocardial infarction. The neutral results in the CIRT trial suggest that future targeted inflammatory therapies should focus on the control of specific inflammatory cytokines or immune cells [16]. One potential strategy is inhibiting the main proinflammatory activation upstream of the NLRP3 inflammasome. NLRP3 inflammasome inhibitors minimized the area of myocardial infarction in animal models [71]. Colchicine, an antirheumatic drug, can cause neutrophil dysfunction and hinder NLRP3 inflammasome evolution in macrophages, resulting in the downregulated expression of the IL-1*β* to IL-6 pathway [19]. The non-double-blind randomized LoDoCo (Low Dose Colchicine) trial enrolled 532 patients with CAD and investigated whether taking colchicine 0.5 mg/day could reduce the risk of cardiovascular events. The results showed that low-cost colchicine could indeed lower the risk of ACS [72]. However, introducing colchicine for the secondary prevention of CAD is difficult because of the modest sample size and defects in trial design that did not involve the use of inflammatory markers as a reference for disease improvement. Recently, the Colchicine Cardiovascular Outcomes (COLCOT) trial showed a promising decline in the primary endpoints in ACS patients with previous myocardial infarction [73]. Although the COLCOT trial verified the potential intervention of RIR independent of aspirin and statins, there were several limitations to this study. Specifically, approximately 20% of patients failed to synchronously follow the trial for various reasons, and most importantly, a portion of the patients showed an increased risk of gastrointestinal intolerance, myelosuppression, and pneumonia following the use of colchicine [73, 74]. The ongoing Colchicine and Spironolactone in Patients with STEMI/SYNERGY Stent Registry (CLEAR-SYNERGY) and Low Dose Colchicine After Myocardial Infarction (LoDoCO2) trials may provide us with more comprehensive data supporting the overall benefit of colchicine [74, 75]. Nevertheless, the CANTOS, CIRT, and COLCOT trials that targeted the NLRP3/IL-1*β*/IL-6 pathway have provided evidence of the potential benefits of targeting proatherogenic immune networks [74].

TNF-*α* inhibitors are also widely used to reduce inflammation and regulate endothelial dysfunction, which may improve the outcome of chronic inflammatory diseases [12]. A systematic review and meta-analysis of 13 cohort studies on rheumatoid arthritis demonstrated that TNF-*α* inhibitors as adjunctive therapy, including disease-modifying antirheumatic drugs (DMARDs), reduced the risk of ACS. However, three randomized controlled trials (RCTs) failed to show any cardiovascular protective effects of TNF-*α* inhibitors [19, 76]. Therefore, the cardiovascular benefits of TNF-*α* inhibitors remain elusive, and large-scale clinical trials are needed to further verify the potential outcomes of TNF-*α* inhibitors in ACS patients. Agents that promote macrophage efflux or enhance efferocytosis showed promising effects on the reduction of ACS events by restricting the expression of netrin-1 and delivering nanoparticles loaded with siRNA to M1 macrophages [8]. CD47, also referred to as the “Do not-Eat-Me” signal, has received great attention in recent years and has been found to be overexpressed in most cancers [77]. CD47-blocking antibodies modified the accumulation of central lipid cores by improving the function of phagocytosis in mice with atherosclerosis [25, 78]. Therefore, the administration of CD47-blocking antibodies is a feasible anti-inflammatory strategy to mitigate the risks of ACS.

Other therapies targeting inflammation have also been investigated. The phospholipase A2 (PLA2) enzymes are key contributors to lipid metabolism and inflammatory activation and are strongly correlated with plaque burden [47, 79]. PLA2 inhibition with varespladib methyl or darapladib showed indistinctive improvements in overall cardiovascular events but rendered patients susceptible to ACS in several large clinical trials [19, 47]. A similar result was observed in large randomized trials of mitogen-activated protein kinase (MAPK) signaling cascade-related targeted therapies [47, 80]. As MAPK activation amplified inflammatory responses, MAPK inhibitors could moderate the systemic or local residual inflammation that leads to ACS [81]. Therefore, their effectiveness as a targeted therapy still needs to be explored.

### 5.2. Targeting Antiatherogenic Immune Modulation to Inhibit Inflammation in ACS

Due to limited atheroprotective autoimmunity, it is often difficult to prevent excessive proatherogenic immune responses. An improvement in the antiatherogenic ability is expected to affect immune homeostasis. Anakinra, an IL-1Ra antagonist, blocks endogenous IL-1*β* and the downstream sequelae and has been used clinically for alleviating rheumatoid diseases. An acute, double-blind trial involving 23 patients with rheumatoid arthritis receiving anakinra (150 mg) or placebo confirmed the significant improvement in biomarkers and vascular and left ventricular function in the anakinra group [82]. Anakinra is expected to improve the outcome of atherosclerosis regarding the provocative results of the CANTOS trial. The administration of anakinra in ACS patients is progressively underway, and positive inflammation-reducing effects have been observed [3]. The results of phase II clinical trials involving investigational drugs for ACS are expected to verify the promising clinical outcomes of anakinra [83].

Recently, T_reg_ cell-based treatments have received great attention. T_reg_ cells induced by specific antigens restored the internal immune environment and reversed atherosclerosis in mice [7, 84]. An alternative proposed strategy involving the purification of natural T_reg_ cells from subjects and their expansion *in vitro* before reinfusion has proven to be effective in the prevention of atherosclerosis progression [7]. The stability of autologous T_reg_ cells during their *in vitro* expansion and after their targeted delivery into hyperactive inflammation sites remains problematic when used for the treatment of human atherosclerosis. Although T_reg_ cell-based treatments alone might not be sufficient to prevent patients from ACS because of the persistent existence of traditional cardiovascular risk factors, optimizing T_reg_ cell-based treatments is a promising approach from a mechanistic perspective. Therefore, T_reg_ cell-based treatments may be used as a promising antiatherogenic adjuvant strategy for the management of ACS.

## 6. Prospective of Therapeutic Strategies Targeting Immune and Inflammation in ACS

Although several studies targeting immune and inflammation in ACS showed improved outcomes of atherosclerosis, the future in this field is full of unknowns. Certain inflammatory pathways or specific immune cells are not the perfect equivalents of systemic inflammatory responses. Fleming et al. proposed the fixed distinction between the “surrogate” and “correlate” of a disease [47, 85]. This conceptual difference indicates that inflammatory diseases may produce several biomarkers that are not the real cause of the ultimate clinical outcome. For example, PLA2 and CRP are highly expressed in ACS and positively correlated with the risk of plaque rupture, but their targeted therapies do not achieve the desired effect. Moreover, if a selected “surrogate” only targets one of the multiple proatherogenic or antiatherogenic pathways of ACS without the intervention of the other active pathways, the clinical endpoints may not be effectively modified [47]. The reason for the success of therapeutic strategies targeting the NLRP3 to IL-1*β* to IL-6 pathway is that these factors serve as perfect representatives that capture abundant upstream inflammatory signals and lead directly to the magnification of the systemic or local inflammation [16]. In addition, some anti-inflammatory agents appear promising and beneficial effects in animals but not in human, such as MAPK inhibitor losmapimod and the recombinant P-selectin glycoprotein ligand-1 (PSGL-1) [47]. One of the most convincing explanations for this is related to the optimal time of drug administration. In animal experiments, these anti-inflammatory agents can be administered immediately after the established severe inflammatory stimulus, whereas it is not practical to initiate them before or when undetected ACS occurs in patients. These pose some challenges for future experimental and human investigation. The successful targeting of immune and inflammation depends not only on the discovery of a more ideal “surrogate” for inflammation but also on the development of an experimental model more similar to humans [47].

To date, studies on targeting proatherogenic inflammation have outnumbered those targeting antiatherogenic inflammation. The clinical benefits observed in most trials investigating the regulation of proatherogenic mechanisms alone were suboptimal. Furthermore, the overwhelming suppression of proatherogenic immunomodulatory effects attenuates systemic immunocompetence and results in multiple infections and malignant tumors [7, 16]. Accumulating evidence indicates that proatherogenic and antiatherogenic immunomodulatory effects are equally important in the complex human immune network, and promising attempts have been made to reinforce antiatherogenic T lymphocyte subsets and other anti-inflammatory cytokines to restore the balance of autoimmune regulation in the treatment of autoimmune diseases, cancer, and allogeneic transplantation [7]. Therefore, efforts should focus on antiatherogenic adjuvant agents in the future development of immunotherapies for ACS.

## 7. Conclusions

ACS has been referred to as an inflammation-related disease regarding its pathogenesis. Understanding the mechanisms of immune and inflammation in ACS will transform risk evaluations and treatment paradigms. The management of RIR beyond traditional guideline-based therapy leads to positive cardiovascular outcomes. Efforts targeting the immune system and inflammation to alleviate the ACS burden have provided promising results. Although they are still being developed, therapies based on anti-inflammation and immune modulation will promote a personalized medicine in the future. Finally, cooperation among cardiologists, oncologists, and rheumatologists is needed to achieve precise prevention and therapy for ACS patients.

## Figures and Tables

**Table 1:** Major published and ongoing clinical studies targeting inflammation therapies in coronary artery disease.

Study	Subjects	Inflammatory target	Therapeutic agent	Median follow-up duration	Primary outcome	Benefit achieved
LoDoCo	Patients with stable CAD	Broad spectrum	Colchicine	3 years	Cardiac arrest, ACS, stroke	Yes [72]
CANTOS	Post-ACS patients with high level of hsCRP	IL-1*β*	Canakinumab	48 months	Cardiovascular death, nonfatal myocardial infarction or stroke	Yes [6]
CIRT	Type 2 diabetes or metabolic syndrome patients with recent ACS	Broad spectrum	Methotrexate	2.3 years	Cardiovascular death, nonfatal myocardial infarction or stroke, hospitalization for emergency revascularization	No [68]
COLCOT	Post-ACS patients	Broad spectrum	Colchicine	22.6 months	Cardiovascular death, resuscitated cardiac arrest, myocardial infarction, stroke, hospitalization for emergency revascularization	Yes [73]
CLEAR-SYNERGY	ACS patients with STEMI/SYNERGY stent	Broad spectrum	Colchicine and spironolactone	2 years	Cardiovascular death, stroke, recurrent myocardial infarction	Ongoing [75]
LoDoCo2	Patients with stable CAD	Broad spectrum	Colchicine	3 years	Cardiovascular death, ACS, stroke	Ongoing [75]
ASSAIL-MI	Patients with first STEMI	IL-6	Tocilizumab	6 months	Myocardial salvage index assessed by CMR 1 week after administration	Ongoing [70]
LATITUDE-TIMI 60	Patients with ACS	Mitogen-activated protein kinase	Losmapimod	12 weeks	Cardiovascular death, myocardial infarction, recurrent angina requiring emergency revascularization	No [80]
VCU-ART3	Patients with ACS	IL-1Ra	Anakinra	12 months	14-day changes in CRP levels, new-onset heart failure, long-term improvement of left ventricular ejection fraction	Ongoing [83]

Footnote. ACS: acute coronary syndrome; CRP: C-reactive protein; CMR: cardiovascular magnetic resonance; LoDoCo: Low-Dose Colchicine; CANTOS: Canakinumab. Anti-Inflammatory Thrombosis Outcome Study; CIRT: Cardiovascular Inflammation Reduction Trial; COLCOT: Colchicine Cardiovascular Outcomes Trial; CLEAR-SYNERGY: Colchicine and Spironolactone in Patients with STEMI/SYNERGY Stent Registry; LoDoCo2: Low Dose Colchicine After Myocardial Infarction; ASSAIL-MI: ASSessing the Effect of Anti-IL-6 Treatment in MI; LATITUDE-TIMI 60: LosmApimod To InhibiT p38 MAP kinase as a therapeUtic target and moDify outcomes after an acute coronary syndrome; VCU-ART3: Virginia Commonwealth University-Anakinra Remodeling Trial-3.
